# Supplementary material for: Polygamous breeding system identified in the distylous genus Psychotria: P. manillensis in the Ryukyu archipelago, Japan
Source: PeerJ. 2021 Nov 10;9:e12318. doi: 10.7717/peerj.12318 (PMC8590391; doi:10.7717/peerj.12318)
Supplement: Supplemental Information 1 [file peerj-09-12318-s001.pdf]

Table S1. Fruit set of *Psychotria manillensis* in five populations of Okinawa and Iriomote Islands, Japan.

		Population	N	*N*	n	Fruit set (%)	Percentage of fruiting plants
**Open pollination**							
**2011**	Okinawa Isl.						
		Sueyoshi	20	60	3138	12.5	100
		Katsuu	17	51	1828	21.9	100
		Oppa	33	99	4719	19.8	88.0
**2012**	Okinawa Isl.						
		Sueyoshi	20	60	4066	16.2	95.0
		Katsuu	23	61	6418	5.1	95.2
		Oppa	23	69	3896	8.2	95.7
	Iriomote Isl.						
		Sonai	20	58	2940	6.6	70.0
		Uehara	14	42	2135	1.8	64.3
**2013**	Okinawa Isl.						
		Sueyoshi	23	69	5064	6.9	100.0
		Katsuu	25	75	9061	12.0	100.0
		Oppa	22	66	6370	4.6	100.0
	Iriomote Isl.						
		Sonai	21	63	3818	3.3	95.2
		Uehara	13	39	1998	2.2	76.9
**Bagging experiment**							
**2012**	Okinawa Isl.						
		Sueyoshi	10	10	444	0.68	-
		Katsuu	11	11	663	0.75	-
		Oppa	18	18	642	1.56	

N, number of plants

N, number of inflorescence

n, number of flowers
