# Supplementary material for: Polygamous breeding system identified in the distylous genus Psychotria: P. manillensis in the Ryukyu archipelago, Japan
Source: PeerJ. 2021 Nov 10;9:e12318. doi: 10.7717/peerj.12318 (PMC8590391; doi:10.7717/peerj.12318)
Supplement: Supplemental Information 2 [file peerj-09-12318-s002.pdf]

Table S2. AIC of GLMM (logit-link, binomial distribution) and results of maximum likelihood test (chi-square) between models.

	Models	Responsible variable	Fixed variable	Random effect	AIC	Maximum likelihood test between models	
						Chisq	P value
Fruit set after cross pollination							
	model.0	fruit set	none	plant id	249	102.07	<0.0001
	model.1	fruit set	stigma types	plant id	151		
Fruit set after self- and cross polination							
	model.0	fruit set	none	plant id + stigma type	257	32.21	<0.0001
	model.1	fruit set	treatment (cross/self)	plant id + stigma type	227		
Fruit set under open pollination							
	model.0	fruit set	none	plant id	6953		
	model.1	fruit set	year	plant id	6809		
	model.2	fruit set	population	plant id	6914		
	model.3	fruit set	year + population	plant id	6780		
Fruit set after bagging and open pollination in 2012							
	model.0	fruit set	none	plant id / population	2113	73.74	<0.0001
	model.1	fruit set	treatment (bag/open)	plant id / population	2041		
