# Supplementary material for: Polygamous breeding system identified in the distylous genus Psychotria: P. manillensis in the Ryukyu archipelago, Japan
Source: PeerJ. 2021 Nov 10;9:e12318. doi: 10.7717/peerj.12318 (PMC8590391; doi:10.7717/peerj.12318)
Supplement: Supplemental Information 3 — Number of visits to an inflorescence during an 8-h observation are shown. Kt, Katsuu population; Op, Oppa population; Su, Sueyoshi population; Ue, Uehara population; So, Sonai population. Observations were performed on July 17 and 18, 2011 and on July 14 and 20, 2012 in the Katsuu population; from July 6 to September 18, 2011 and on July 14, 2012 in the Oppa population; on July 9 and 27, 2011, on June 19 and July 1, 2012 in the Sueyoshi population; and on June 2, 3, 15, 16, and 17, 2011 and on June 13, 2013 in the Sonai and Uehara populations. [file peerj-09-12318-s003.pdf]

Table S3. Flower visitors of *Psychotria manillensis* on Okinawa and Iriomote Islands, the Ryukyu Islands, Japan, in 2011-2013.

		Population				
		Okinawa			Iriomote	
Family	Species	Kt	Op	Su	Ue	So
**HYMENOPTERA**						
Apidae	*Amegilla dulcifera*		1	1		
	A. florea					5
	Apis mellifera	2	2			
Halictidae	*Lasioglossum* sp.	1	2	4		
Icheumonidae	sp.		1			
Scoliidae	*Megacampsomeris prismatica*			1		
Vespidae	*Anterhynchium flavomarginatum*	1				
	Pararrhynchium ishigakiense					1
	Polistes formosanus				22	16
	P. rothneyi	23	26	59	32	26
	Vespa affinis				7	7
	V. analis	33	29	1	31	22
	V. ducalis				7	5
**DIPTERA**						
Ephydridae	sp.	1				
Calliphoridae	*Hemipyrellia* sp.	2			1	
	Lucilia papuensis	1			1	
	L. porphyrina	12	22	10	9	4
Muscidae	*Dichaetomyia flavipolis*	3	7	*5*		
	D. watasei	3	1			
	Stomoxys calcitrans	1				
Sarcophagidae	*Sarcophaga antilope*		1			
	S. kanakovi		1			
	sp.	1				
Syrphidae	*Allobaccha nubilipennis*	16	3	1	1	
	sp.		2			
Tachinidae	sp.		1			
**LEPIDOPTERA**						
Crambidae	sp. 1		1			
	sp. 2		2			
	sp. 3		1			
Lycaenidae	*Lampides boeticus*	1				
Nymphalidae	*Athymaperius perius*		1			
Papilionidae	*Papilio memnon*	2				
	Papilio okinawaensis	1				
	Papilio polytes	4	1			
Sphingidae	*Macroglossum pyrrhosticta*		2			
**COLEOPTERA**						
Chrysomelidae	*Altica cyanea*	1				
**HEMIPTERA**						
Largidae	*Physopelta cincticollis*		1			
**ORTHOPTERA**						
Gryllidae	*Cardiodactylus guttulus*	1	1		1	1
	Gryllodes sigillatus					2

Number of visits to an inflorescence during an 8-h observation are shown. Kt, Katsuu population; Op, Oppa population; Su, Sueyoshi population; Ue, Uehara population; So, Sonai population. Observations were performed on July 17 and 18, 2011 and on July 14 and 20, 2012 in the Katsuu population; from July 6 to September 18, 2011 and on July 14, 2012 in the Oppa population; on July 9 and 27, 2011, on June 19 and July 1, 2012 in the Sueyoshi population; and on June 2, 3, 15, 16, and 17, 2011 and on June 13, 2013 in the Sonai and Uehara populations.
